# The Lab4P Consortium of Probiotics Attenuates Atherosclerosis in LDL Receptor Deficient Mice Fed a High Fat Diet and Causes Plaque Stabilization by Inhibiting Inflammation and Several Pro‐Atherogenic Processes

**DOI:** 10.1002/mnfr.202100214

**Published:** 2021-07-19

**Authors:** Victoria L. O'Morain, Yee‐Hung Chan, Jessica O. Williams, Reem Alotibi, Alaa Alahmadi, Neil P. Rodrigues, Sue F. Plummer, Timothy R. Hughes, Daryn R. Michael, Dipak P. Ramji

**Affiliations:** ^1^ Cardiff School of Biosciences Cardiff University Sir Martin Evans Building, Museum Avenue Cardiff CF10 3AX UK; ^2^ Systems Immunity Research Institute, School of Medicine Cardiff University Cardiff CF14 4XN UK; ^3^ European Cancer Stem Cell Research Institute, Cardiff School of Biosciences Cardiff University Hadyn Ellis Building, Maindy Road Cardiff CF24 4HQ UK; ^4^ Cultech Limited Unit 2 Christchurch Road, Baglan Industrial Park Port Talbot SA12 7BZ UK

**Keywords:** atherosclerosis, foam cells, gene expression, LDL receptor deficient mice, probiotics

## Abstract

**Scope:**

Previous studies show that Lab4 probiotic consortium plus *Lactobacillus plantarum* CUL66 (Lab4P) reduces diet‐induced weight gain and plasma cholesterol levels in C57BL/6J mice fed a high fat diet (HFD). The effect of Lab4P on atherosclerosis is not known and is therefore investigated.

**Methods and Results:**

Atherosclerosis‐associated parameters are analyzed in LDL receptor deficient mice fed HFD for 12 weeks alone or supplemented with Lab4P. Lab4P increases plasma HDL and triglyceride levels and decreases LDL/VLDL levels. Lab4P also reduces plaque burden and content of lipids and macrophages, indicative of dampened inflammation, and increases smooth muscle cell content, a marker of plaque stabilization. Atherosclerosis arrays show that Lab4P alters the liver expression of 19 key disease‐associated genes. Lab4P also decreases the frequency of macrophages and T‐cells in the bone marrow. In vitro assays using conditioned media from probiotic bacteria demonstrates attenuation of several atherosclerosis‐associated processes in vitro such as chemokine‐driven monocytic migration, proliferation of monocytes and macrophages, foam cell formation and associated changes in expression of key genes, and proliferation and migration of vascular smooth muscle cells.

**Conclusion:**

This study provides new insights into the anti‐atherogenic actions of Lab4P together with the underlying mechanisms and supports further assessments in human trials.

## Graphical abstract

The Lab4P consortium of probiotics attenuates atherosclerosis in LDL receptor deficient mice fed a high fat diet and prevents multiple processes associated with disease progression in vitro. Upward arrow, increase; downward arrow, decrease. LDLr^−/−^, low density lipoprotein deficient mice.

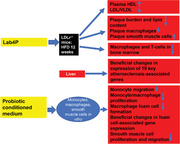

## 1 Introduction

Atherosclerosis, a disease of the vasculature associated with inflammation and lipid accumulation, is the principal cause of cardiovascular disease.^[^ ^1^, ^2^, ^3^ ^]^ Current therapies against atherosclerosis such as statins are not fully effective and many promising pharmaceutical leads from various drug discovery and other screening programs have failed because of safety concerns and/or off‐target effects.^[^ ^1^, ^2^ ^]^ Substantial interest has therefore been fuelled in the use of natural products in the prevention and treatment of atherosclerosis, possibly as adds‐on with pharmaceutical agents.^[^ ^1^, ^2^ ^]^

Probiotic bacteria have many health benefits, including regulation of atherosclerosis‐associated risk factors.^[^ ^2^ ^]^ However, the molecular mechanisms underlying the cardio‐protective actions of probiotics are poorly understood. We have previously shown the cholesterol lowering ability of *Lactobacillus plantarum* CUL66 in Caco‐2 enterocytes via modulation of pathways associated with the metabolism of this sterol.^[^ ^4^ ^]^ In an acute study in C57BL/6J mice fed a high fat diet (HFD), the Lab4 consortium of probiotics, composed of *Lactobacillus acidophilus* CUL21 (NCIMB 30156) and CUL60 (NCIMB 30157), *Bifidobacterium bifidum* CUL20 (NCIMB 30153) and *Bifidobacterium animalis* subsp*. lactis* CUL34 (NCIMB 30172) in combination with *L. plantarum* CUL66 (called Lab4P hereafter) reduced plasma levels of total cholesterol and suppressed diet‐induced weight gain.^[^ ^5^ ^]^ More recently, Lab4P was shown to reduce body weight and improve well‐being in a randomized control trial of overweight and obese adults.^[^ ^6^ ^]^ However, the effects of Lab4P on atherosclerosis are not known and were investigated using LDL receptor deficient (LDLr^−/−^) mice fed HFD for 12 weeks. Lab4P produced beneficial changes in the plasma cholesterol profile, plaque burden, inflammation and a marker of stability, liver expression of several atherosclerosis‐associated genes and bone marrow cell populations. The use of conditioned medium (CM) from probiotic bacteria demonstrated beneficial regulation of several atherosclerosis‐associated processes in vitro and provided mechanistic insights.

## 2 Results

### 2.1 Lab4P Increases Spleen Weight and Produces Many Beneficial Changes in the Plasma Lipid Profile

Lab4P had no significant effect on high fat diet (HFD)‐induced weight gain or final weight of mice or that for subcutaneous fat, gonadal fat, liver, heart or thymus (**Table** **1**). However, a significant increase was seen in the weight of spleen (*p =* 0.034). Lab4P also produced significant decrease in plasma levels of LDL/VLDL (*p =* 0.048) and significant increase in plasma levels of HDL (*p =* 0.039) and triglyceride (TG) (*p =* 0.019) associated with significant reduction in the ratio of LDL/VLDL:HDL (*p =* 0.002) and total cholesterol (TC):HDL (*p =* 0.031) and significant increase in the TC:LDL/VLDL ratio (*p =* 0.044).

**Table 1:** The effect of Lab4P supplementation on organ weights and plasma lipid profile in LDLr^−/–^ mice fed HFD

	Control	Lab4P	*p* Value
Mouse weight [g]	35.9 ± 1.4	36.6 ± 1.5	0.764
Weight gain [g]	12.5 ± 1.6	11.7 ± 1.4	0.715
Subcutaneous fat weight [g]	1.39 ± 0.24	1.33 ± 0.22	0.853
Gonadal fat weight [g]	1.73 ± 0.26	1.67 ± 0.25	0.875
Liver weight [mg]	145.5 ± 6.6	147.5 ± 7.9	0.842
Spleen weight [mg]	110.8 ± 3.1	125.4 ± 5.7	0.034
Heart weight [mg]	181.5 ± 5.2	185.0 ± 3.9	0.594
Thymus weight [mg]	47.3 ± 3.7	50.0 ± 4.0	0.630
LDL/VLDL [mg dL^‐1^]	921.8 ± 62.4	743.3 ± 59.6	0.048
HDL [mg dL^‐1^]	128.9 ± 11.1	169.9 ± 15.1	0.039
TC [mg dL^‐1^]	1993 ± 68	2159 ± 94	0.161
FC [mg dL^‐1^]	633.9 ± 48.1	708.2 ± 49.3	0.301
CE [mg dL^‐1^]	1272 ± 106.8	1386 ± 89.3	0.423
TG [mg dL^‐1^]	339.6 ± 28.2	446.0 ± 32.2	0.019
LDL/VLDL:HDL	7.5 ± 0.6	4.5 ± 0.5	0.002
TC:HDL	17.6 ± 1.5	12.9 ± 1.2	0.031
TC:LDL/VLDL	2.2 ± 0.2	2.9 ± 0.3	0.044

Data represents the mean ± SEM from 30 mice (control n = 15; Lab4P n = 15). Statistical analysis was performed using an unpaired Student's *t*‐test. Values of *p* are stated and considered significant when *p* ≤ 0.05.

Abbreviations: CE, cholesterol esters; FC, free cholesterol; HDL, high‐density lipoprotein; LDL, low‐density lipoprotein; TC, total cholesterol; TG, triglycerides; VLDL, very low‐density lipoprotein.

### 2.2 Lab4P Produces Beneficial Changes in Plaque Parameters in the Aortic Root

**Figure 1:**
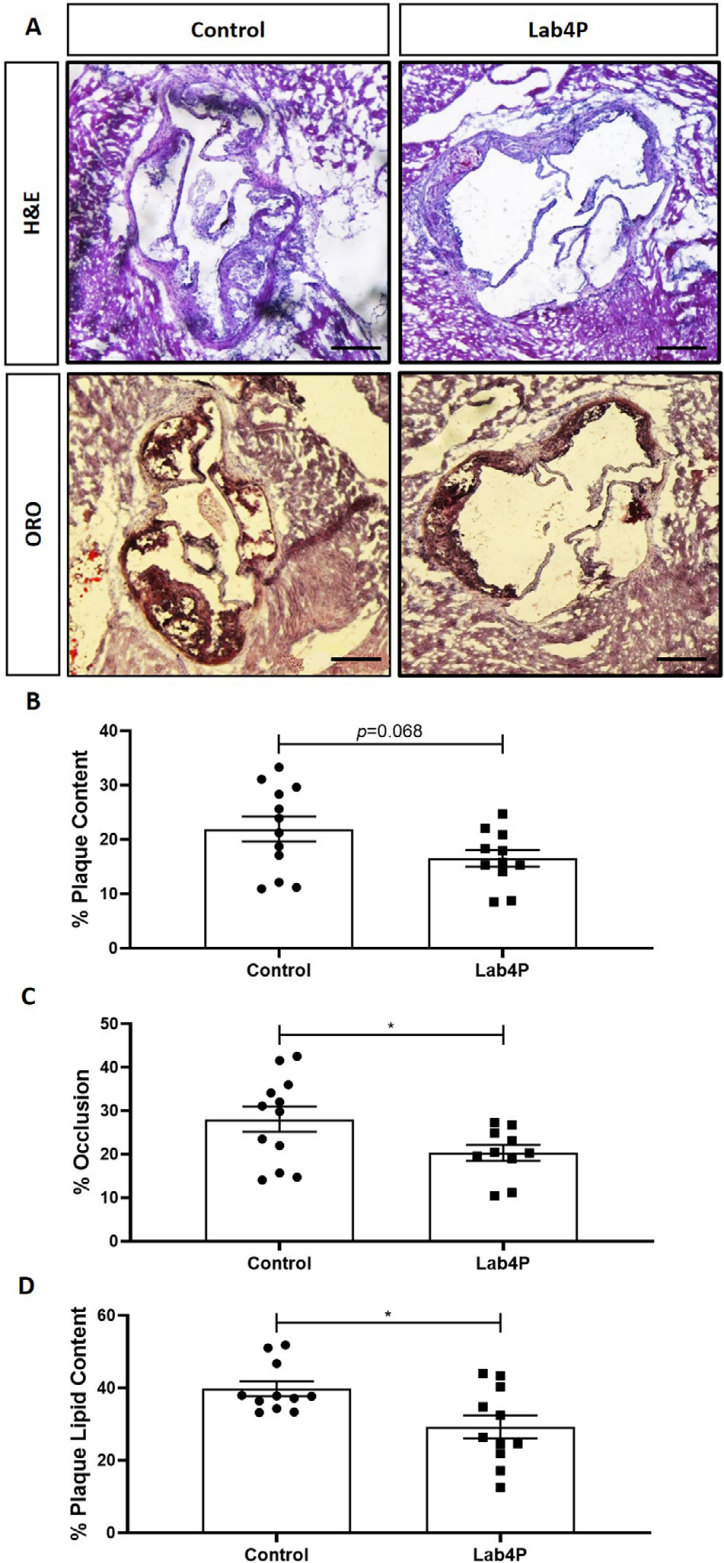
Lab4P produces a decrease in plaque burden and lipid content in LDLr^−/−^ mice fed HFD. Sections from the aortic root of mice fed HFD for 12 weeks (Control) or HFD supplemented with Lab4P (Lab4P) were stained with hematoxylin & eosin (H&E) or oil red O (ORO). (A) Shows representative images from this staining (x40 magnification; scale bar of 50 µm). Plaque content (B), occlusion (C) and plaque lipid content (D) were determined using the Image J software. Data are mean ± SEM (n = 12 for Control and 11 for Lab4P). Statistical analysis was carried out using an unpaired Student's *t* test (*p ≤ 0.05).

**Figure 2:**
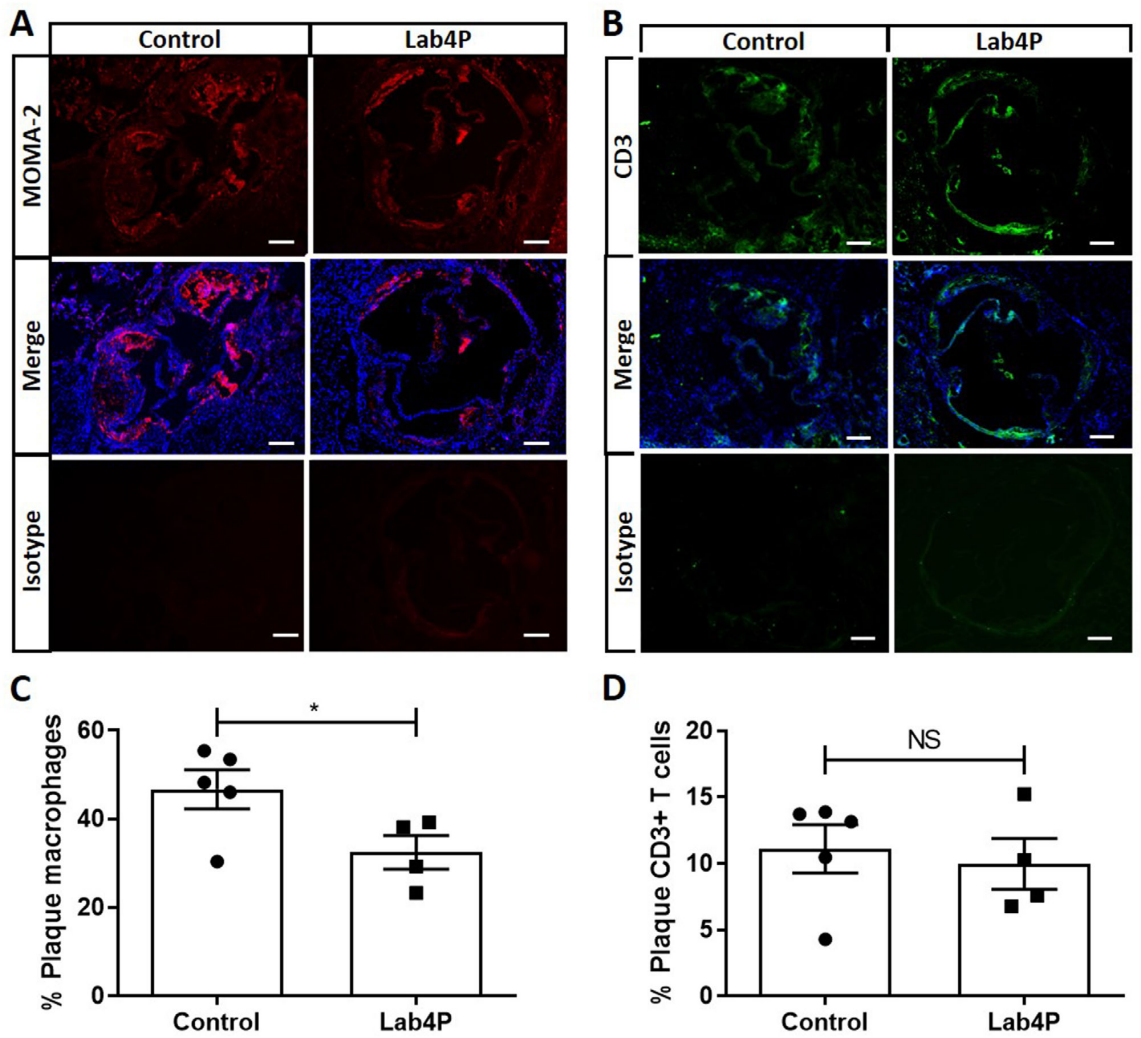
Lab4 produces a significant decrease in plaque macrophage content in LDLr^−/−^ mice fed HFD. Immunohistochemistry (IHC) of sections from the aortic root of mice fed HFD for 12 weeks (Control) or HFD supplemented with Lab4P (Lab4P) were stained using the MOMA‐2 antibody or CD3 antibody (both mounted with DAPI) or appropriate isotype control antibodies and images taken by fluorescent microscopy. Representative images are shown for control and Lab4P groups for MOMA‐2 staining (MOMA‐2), MOMA‐2 and DAPI staining (Merge) and IgG isotype control (Isotype) (A) or CD3 staining (CD3), CD3 and DAPI staining (Merge) and IgG isotype control (Isotype) (B) (scale bars 50 µm; red, MOMA‐2 AF‐488; green, CD3 AF‐594; blue, DAPI). The plaque content of macrophages (C) or CD3+ T cells (D) were determined using the Image J software. Data are mean ± SEM (n = 5 for Control and 4 for Lab4P). Statistical analysis was carried out using an unpaired Student's *t* test (*p ≤ 0.05).

**Figure 3:**
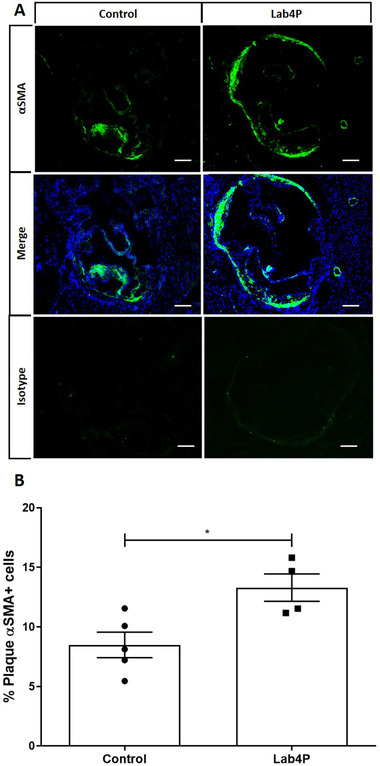
Lab4 produces a significant increase in plaque content of smooth muscle cells (αSMA‐positive) in LDLr^−/−^ mice fed HFD. IHC of sections from the aortic root of mice fed HFD for 12 weeks (Control) or HFD supplemented with Lab4P (Lab4P) were stained using the αSMA antibody, mounted with DAPI, or appropriate isotype control antibodies and images taken by fluorescent microscopy. Representative images are shown for control and Lab4P groups for αSMA staining (αSMA), αSMA and DAPI staining (Merge) and IgG isotype control (Isotype) (A). The plaque content of αSMA‐positive smooth muscle cells was determined using the Image J software. Data are mean ± SEM (n = 5 for Control and 4 for Lab4P). Statistical analysis was carried out using an unpaired Student's t test (*p ≤ 0.05).

Lab4P produced a trend towards decrease in % plaque content (*p =* 0.068) and significant reduction in percentage occlusion (plaque/vessel x 100) and plaque lipid content (*p =* 0.043 and *p =* 0.019, respectively) (**Figure** **1**). Lab4P also produced significant decrease in plaque macrophage (*p =* 0.050) but not CD3 T cells (**Figure** **2**) and caused significant increase in α‐smooth muscle cell actin (αSMA) staining, indicative of plaque stabilization (*p =* 0.019) (**Figure** **3**).

### 2.3 Lab4P Produces Beneficial Changes in Liver Expression of Key Atherosclerosis‐Associated Genes

**Figure 4:**
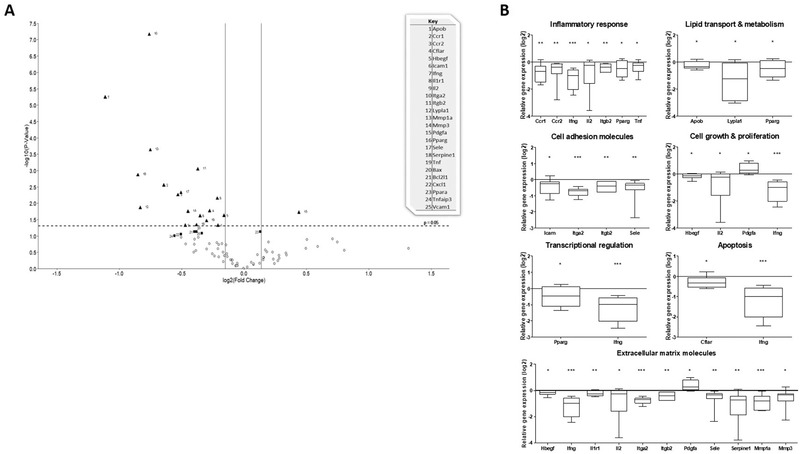
Volcano plot and class of genes whose liver expression was altered by Lab4P. The liver expression of atherosclerosis‐associated genes was assessed in LDLr^−/−^ mice fed with either HFD (control group) or HFD supplemented with Lap4P for 12 weeks. (A) Volcano plot where the data are presented as mean fold‐change in gene expression, normalized to five housekeeping genes, in the Lab4P group compared to the control. The log2 fold‐change in gene expression is represented on the x‐axis with the y‐axis showing the ‐log10 of the p value. A p value of 0.05 (dashed line) and fold change of ±10% (solid lines) are indicated. Black triangles indicate genes showing significant changes in expression and black squares show genes with a trend towards expression (*p* between 0.05 and 0.100). (B) Genes showing significant change in expression sorted into related groups based on their function. Data are presented as box and whisker plots of log2 fold‐change in gene expression in the Lab4P group relative to the control, where whiskers represent min to max fold‐change. Statistical analysis was performed using an unpaired Student's *t*‐test (*p < 0.05, **p < 0.01, ***p < 0.001).

ORO staining of liver sections showed that Lab4P produces a decrease in lipid content (Figure S1, Supporting Information). As liver gene expression has a major impact on changes in metabolism and inflammation during atherosclerosis,^[^ ^7^, ^8^ ^]^ the effect of Lab4P on this parameter was investigated using Atherosclerosis RT² Profiler PCR Array. Fold‐changes in liver gene expression together with the *p*‐value for each gene are given in Table S4, Supporting Information whereas **Figure** **4**A shows a volcano plot for all gene expression changes. Expression of 19 genes was significantly affected by Lab4P with a further 6 demonstrating a trend (*p* values between 0.050 and 0.100).

Genes were grouped into related classes on their function and Figure S2 shows heat maps and cluster analysis of gene expression. Significant changes in expression in each group are shown graphically in Figure 4B (some genes have pleiotropic functions and hence included in more than one group). For genes implicated in control of inflammation, expression of *chemokine C‐C motif receptor 1* (*Ccr1*; *p =* 0.003), *Ccr2* (*p =* 0.006), *interferon‐γ* (*Ifng*; *p <* 0.001), *interleukin (IL)‐2* (*Il2*; *p =* 0.048), *integrin β2* (*Itgb2*; *p =* 0.001), *peroxisome proliferator activated receptor (Ppar) γ* (*Pparg*; *p =* 0.046) and *tumor necrosis factor* (*Tnf*; *p =* 0.035) were significantly reduced in the Lab4P group. Similarly, the expression of three genes involved in lipid transport and metabolism [*apolipoprotein B* (*Apob*; *p =* 0.048), *lysophospholipase 1* (*Lypla1*; *p =* 0.014) and *Pparg* (see above)] was decreased. Expression levels of several genes involved in cell adhesion were also significantly reduced [*intercellular adhesion molecule‐1* (*Icam*; *p =* 0.025), *integrin α2* (*Itga2*; *p <* 0.001), *Itgb2* (see above) and *selectin, endothelial cells* (*Sele*; *p =* 0.005)]. For genes involved in cell growth and proliferation, the expression of *heparin‐binding EGF‐like growth factor* (*Hbegf*; *p =* 0.024), *Il2* (see above) and *Ifng* (see above) was significantly decreased while that for *platelet derived growth factor α* (*Pdgfa*; *p =* 0.019) was increased. Expression of genes involved in transcription regulation (*Pparg* and *Ifng*; see above) and apoptosis [*CASP8 and FADD‐like apoptosis regulator* (*Cflar*; *p =* 0.017) and *Ifng* (see above)] were also significantly decreased. Finally, the largest group of genes showing significant changes in expression were those for extracellular matrix molecules, where expression of *Hbegf* (see above), *Infg* (see above), *interleukin 1 receptor, type I* (*Il1r1*; *p* = 0.007), *Il2* (see above), *integrin α2* (*Itga2*; *p <* 0.001), *Itgb2* (see above), *Sele* (see above), *serine (or cysteine) peptidase inhibitor, clade B, member 1* (*Serpine1*; *p =* 0.001), *matrix metallopeptidase 1a* (*Mmp1a p <* 0.001) and *matrix metallopeptidase 3* (*Mmp3*; *p =* 0.018) was decreased and that for *Pdgfa* (see above) increased. Lab4P also produced trend towards reduction of five genes: *BCL2‐associated X protein* (*Bax*; *p =* 0.073) and *BCL2‐like 1*(*Bcl2l1*; *p =* 0.074) implicated in control of apoptosis; *chemokine (C‐X‐C motif) ligand 1* (*Cxcl1*; *p =* 0.087) and *vascular cell adhesion molecule 1* (*Vacm1*; *p* = 0.082) implicated cell adhesion; and *tumor necrosis factor, alpha‐induced protein 3* (*Tnfaip3*; *p =* 0.098). The expression of the transcriptional regulator *Pparα* (*Ppara*) showed a trend towards increase (*p =* 0.074).

### 2.4 Lab4P Produces Beneficial Changes in Bone Marrow Cell Populations

**Figure 5:**
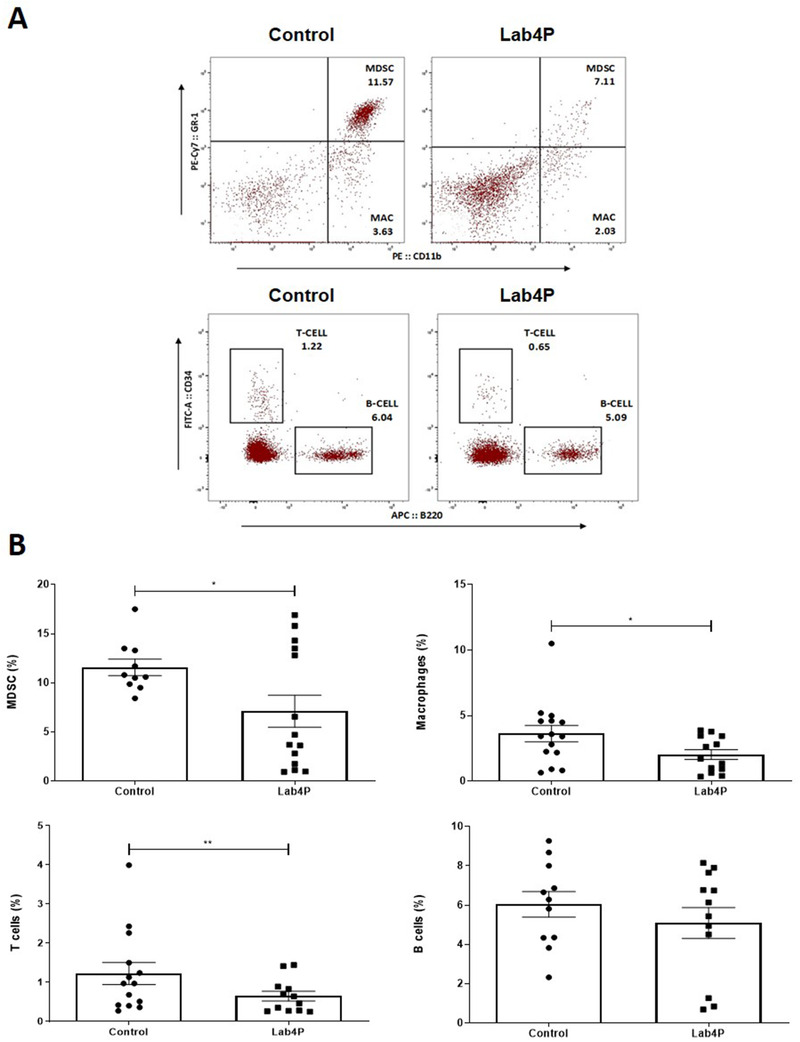
The effect of Lab4P supplementation on bone marrow lineage differentiated cell populations. Immunophenotyping of bone marrow cell populations from LDLr^−/−^ mice was performed following 12 weeks feeding with either HFD (control; n = 15) or HFD supplemented with Lab4P (probiotic; n = 15). (A) Representative flow plots showing lineage positive cell populations in control and Lab4P groups. Gating shows position of populations representing MDSC (GR‐1^+^CD11b^+^), monocytes/macrophages (MAC; GR‐1^−^CD11b^+^), T Cells (CD3^+^B220^−^) and B Cells (CD3^−^B220^+^). (B) The graphs show the frequency of MDSC, monocyte/macrophage (Macrophages), T cell and B cell populations as indicated. The data are mean ± SEM with statistical analysis performed using an unpaired Student's t‐test (*p < 0.05, **p < 0.01).

The levels of stem, progenitor and mature cell populations within the bone marrow changes markedly following HFD feeding and atherogenesis.^[^ ^9^ ^]^ Lab4P produced no significant changes in total cell counts in the bone marrow (Figure S3A, Supporting Information). For lineage positive differentiated cells, Lab4P produced significant reduction in the frequency of MDSC (*p =* 0.041), monocytes/macrophages (*p =* 0.046) and T cells (*p =* 0.005) without affecting B cells (**Figure** **5**). For stem cell populations, Lab4P produced no changes in the frequency of haematopoietic progenitor cell (HPC) I or multipotent progenitor (MPP) cell populations but increased that of HPC II and haematopoietic stem cell (HSC) (*p =* 0.048 and *p =* 0.034, respectively) (Figure S3B, Supporting Information). Lab4P produced no changes in progenitor common lymphoid progenitor (CLP) populations (Figure S3C, Supporting Information).

### 2.5 Conditioned Medium from Lab4 or *L. plantarum* CUL66 Produces Many Beneficial Changes in Atherosclerosis‐Associated Processes in Monocytes, Macrophages and Smooth Muscle Cells In Vitro

In vitro assays were performed to delineate mechanisms underlying beneficial actions of Lab4P seen in LDLr^−/−^ mice in vivo and to determine any potential differences in actions of the Lab4 consortium and *L. plantarum* CUL66 (called Lab4 and CUL66, respectively, hereafter). CM normalized to protein concentration, to rule out any batch specific differences, was used. The CM at maximum concentration used in experiments (corresponding to lowest concentration from different batches) had no detrimental effect on the viability of all cells used (Figure S4, Supporting Information).

**Figure 6:**
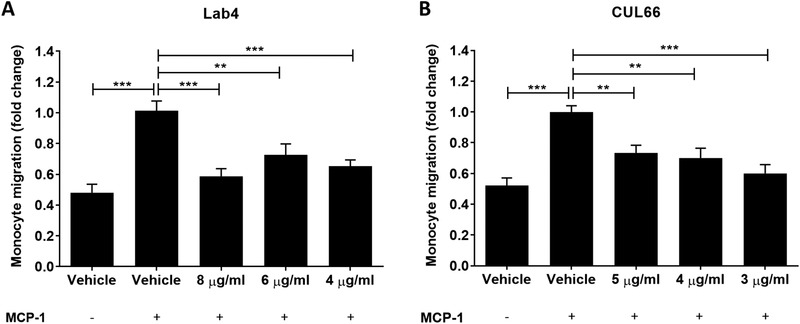
MCP‐1 driven migration of human monocytes was attenuated by different concentrations of CM from Lab4 or CUL66. Migration assays were carried out on THP‐1 monocytes as described in Section 4 using Lab4 CM at concentrations of 8, 6, and 4 µg mL^‐1^ (A) or CUL66 CM at concentrations of 5, 4, and 3 µg mL^‐1^ (B). Cells treated with vehicle but in the absence of MCP‐1 were also included for comparative purposes. Migration was determined as a percentage of the total input cells and displayed as fold‐change in migration relative to the Vehicle control which was arbitrarily set to 1. Data are presented as mean ± SEM from five (A) or three (C) independent experiments. Statistical analysis was performed using a one‐way ANOVA with Dunnett (2‐sided) post‐hoc test where **p < 0.01 and ***p < 0.001.

Lab4P‐mediated decrease in plaque macrophage content in vivo (Figure 2) could be because of reduced chemokine‐driven monocyte recruitment.^[^ ^3^, ^10^ ^]^ CM from both Lab4 and CUL66 at three different concentrations attenuated the MCP‐1 driven monocytic migration seen in vehicle‐treated cells (8 and 5 µg mL^‐1^ corresponded to the lowest concentration of Lab4 and CUL66 obtained in different batches) (**Figure** **6**).

**Figure 7:**
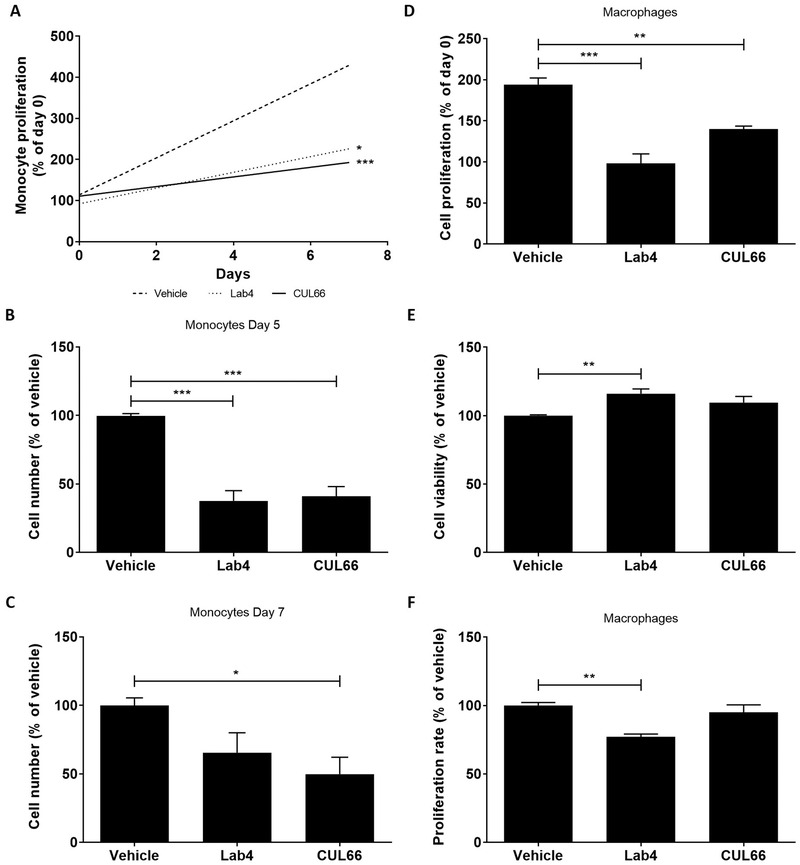
Probiotic CM attenuates the proliferation of human monocytes and macrophages. (A‐C) THP‐1 monocytes (1 × 10^6^ cells) were treated with either the vehicle control or CM from Lab4 (8 µg mL^‐1^) or CUL66 (5 µg mL^‐1^) and cell number counted daily for 7 days. (A) Linear regression of percentage change in cell number from three independent experiments. (B‐C) Cell numbers are displayed as a percentage relative to the Vehicle control at day 5 (B) and day 7 (C) with vehicle control arbitrarily assigned as 100%. (D‐F) THP‐1 macrophages were treated for 48 h with either the vehicle control or Lab4 (8 µg mL^‐1^) or CUL66 (5 µg mL^‐1^) CM. Change in proliferation from day 0 was determined as a percentage relative to the vehicle control (D), cell viability was determined as a percentage to the vehicle control (arbitrarily assigned as 100%) (E) and the proliferation rate was determined using the BrdU Labeling and Detection Kit (F) with the values obtained in vehicle‐treated cells arbitrarily assigned as 100%. In all cases, data are mean ± SEM from three independent experiments with statistical analysis performed using a one‐way ANOVA and Dunnett (2‐sided) post‐hoc test where *p < 0.05, **p < 0.01 and ***p < 0.001.

Changes in monocyte/macrophage proliferation is implicated in regulation of its content in atherosclerotic plaques.^[^ ^11^ ^]^ Human THP‐1 monocytes, which can be differentiated into macrophages using phorbol 12‐myristate 13‐acetate (PMA), were used as they demonstrate conservation of responses relevant to atherosclerosis seen in primary cultures and in vivo.^[^ ^10^, ^12^ ^]^ For monocytes, cells treated with either vehicle or probiotic CM were counted daily for 7 days and proliferation determined as the percentage change in cell number. Linear regression analysis showed significant reduction in monocyte proliferation over the 7‐day period when treated with CM from Lab4 (*p* < 0.05) or CUL66 (*p* < 0.001) (**Figure** **7**A). Changes in cell numbers were also assessed on individual days 2, 5, and 7 and proliferation determined as percentage change relative to vehicle control. No significant changes were seen at day 2 (data not shown) but a reduction was observed with CM from Lab4 and CUL66 that reached significance on day 5 for Lab4 (*p <* 0.001) and both days 5 and 7 for CUL66 (*p <* 0.001 and *p =* 0.023, respectively) (Figure 7B,C).

For THP‐1 macrophages, cellular proliferation was first assessed using crystal violet and to control for any changes in cell viability, the LDH assay was also performed simultaneously. Lab4 or CUL66 CM significantly attenuated proliferation of THP‐1 macrophages (*p <* 0.001 for Lab4 and *p =* 0.001 for CUL66) (Figure 7D) without decreasing cell viability (Figure 7E). 5‐Bromo‐2′‐deoxyuridine (BrdU) incorporation assays showed that Lab4 CM significantly reduced rate of proliferation (*p =* 0.002) whereas no significant changes were observed with CUL66 (Figure 7F).

**Figure 8:**
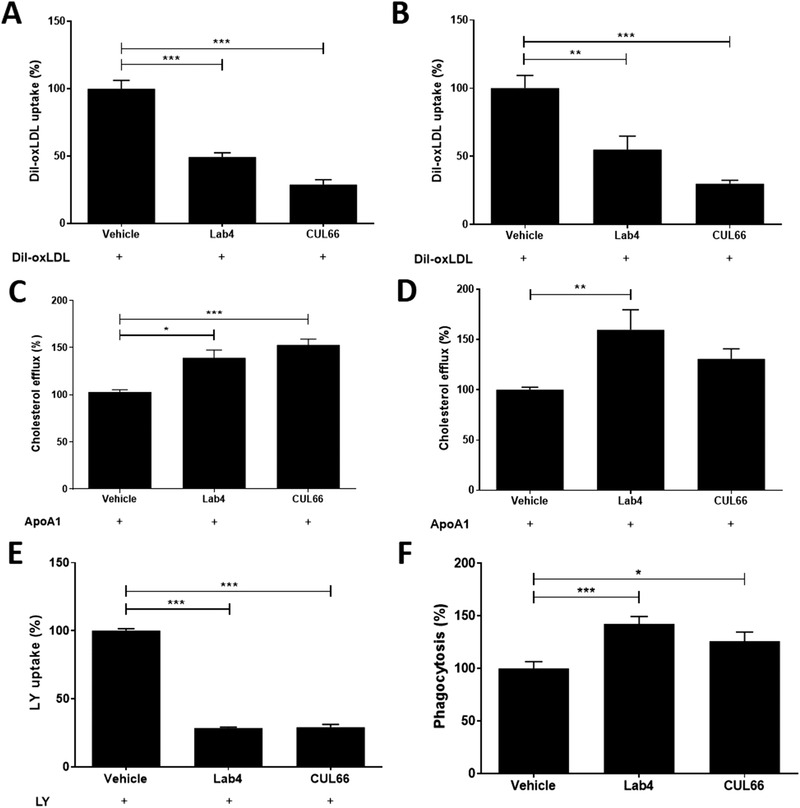
Probiotic CM beneficially regulates several processes in macrophage foam cell formation. Dil‐oxLDL uptake (A‐B), cholesterol efflux (C‐D), LY uptake (E) and phagocytosis (F) was carried out in THP‐1 macrophages (A, C, E‐F) or HMDM (B, D) treated with vehicle or CM from Lab4 (8 µg mL^‐1^) or CUL66 (5 µg mL^‐1^) as described in Section 4. Data are mean ± SEM from three independent experiments with values from vehicle‐treated cells arbitrarily assigned as 100%. Statistical analysis was performed using a one‐way ANOVA with Dunnett (2‐sided) post‐hoc test (A‐E) or Dunnett T3 post‐hoc test (F) (*p < 0.05, **p < 0.01 and ***p < 0.001).

Macrophage foam cell formation is a critical event in atherogenesis controlled by several processes: uptake of oxLDL by receptor‐mediated endocytosis, cholesterol efflux from foam cells, macropinocytosis and phagocytosis.^[^ ^13^ ^]^ Uptake of Dil‐labeled oxLDL was significantly attenuated by CM from both Lab4 and CUL66 (**Figure** **8**A; *p <* 0.001 in both cases). To rule out possibility that the decrease was due to THP‐1 cell line, experiments were performed in primary human monocyte‐derived macrophages (HMDM) where Dil‐oxLDL uptake was also significantly inhibited by Lab4 (*p =* 0.001) and CUL66 (*p <* 0.001) CM (Figure 8B). In contrast, significant increase in cholesterol efflux was observed in THP‐1 macrophage foam cells treated with CM from Lab4 or CUL66 (Figure 8C; *p =* 0.013 and *p <* 0.001, respectively). Lab4 CM also increased cholesterol efflux in primary HMDM (*p* = 0.006) whereas CM from CUL66 produced a non‐significant increase (Figure 8D). CM from Lab4 and CUL66 significantly attenuated macropinocytosis (*p <* 0.001 in both cases) and significantly increased phagocytosis (*p <* 0.001 and *p =* 0.025, respectively) (Figure 8E,F). In the case of macropinocytosis, the assay was also performed using several concentrations of CM and significant reduction was observed in all cases (Figure S5, Supporting Information). Finally, oxidation of LDL by reactive oxygen species (ROS) is also a critical initiator of foam cell formation during atherosclerosis^[^ ^13^ ^]^ but CM from both Lab4 and CUL66 increased tert‐butyl hydroperoxide (TBHP)‐induced ROS production in THP‐1 macrophages (Figure S6, Supporting Information).

**Figure 9:**
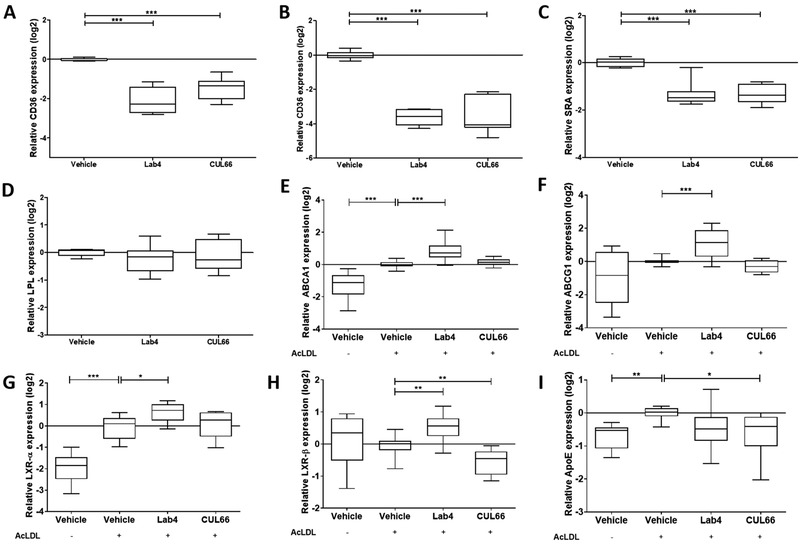
Probiotic CM produces beneficial changes in the expression of several key genes implicated in the control of macrophage foam cell formation. THP‐1 macrophages (A, C, D), HMDM (B) or THP‐1 macrophages converted into foam cells (E‐I) were treated for 24 h with either vehicle control or CM from Lab4 (8 µg mL^‐1^) or CUL66 (5 µg mL^‐1^). Gene expression was determined by qPCR using the ΔΔCT method. Data are presented as box and whisker plots of log2 fold‐change in gene expression relative to the vehicle control, where whiskers represent min to max fold‐change from three independent experiments. Statistical analysis was performed using a one‐way ANOVA with Dunnett (2‐sided) (A‐C, G‐H) or Dunnett T3 (B, E‐I) post‐hoc test (*p < 0.05, **p < 0.01 and ***p < 0.001).

CD36 is a key scavenger receptor in uptake of oxLDL^[^ ^13^ ^]^ and its mRNA expression was significantly attenuated by CM from Lab4 and CUL66 in both THP‐1 macrophages and HMDM (**Figure** **9**A,B; *p <* 0.001 in all cases). Subsequent studies focused on THP‐1 macrophages where the effects of CM on two key genes implicated in the uptake of modified LDL by macrophages (*SRA* and *LPL*) and, following their conversion to foam cells by incubation with acetylated LDL, five genes involved in cholesterol efflux (*ABCA1*, *ABCG1*, *LXRα*, *LXRβ*, and *ApoE*)^[^ ^13^ ^]^ were investigated. CM from Lab4 and CUL66 significantly attenuated expression of *SRA* (*p <* 0.001 in both cases) whereas no effect was seen for LPL (Figure 9C,D). Lab4 CM produced significant increase in expression of *ABCA1* (*p <* 0.001), *ABCG1* (*p <* 0.001) and *LXRα* (*p =* 0.013) whereas CM from CUL66 had no effect (Figure 9E‐G). Lab4 CM also significantly increased expression of *LXRβ* gene (*p =* 0.007) whereas CUL66 CM decreased this (*p =* 0.008) (Figure 9H). For *ApoE* gene, Lab4 CM had no effect whereas CUL66 CM produced a significant reduction (Figure 9I, *p =* 0.011).

**Figure 10:**
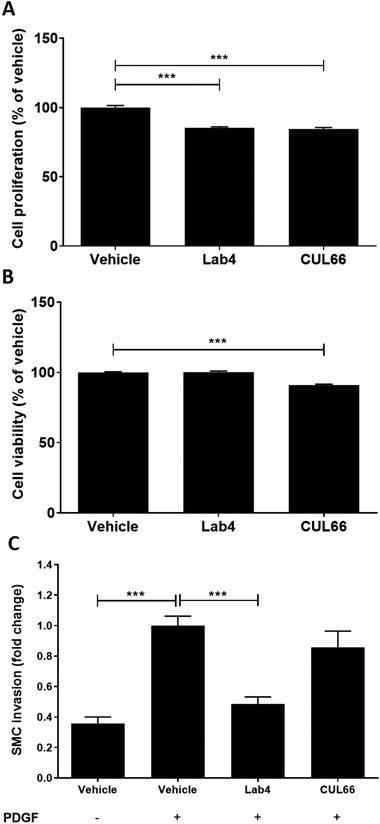
Probiotic CM modulates proliferation and migration of HASMC. The effect of vehicle or CM from Lab4 (8 µg mL^‐1^) or CUL66 (5 µg mL^‐1^) on cell proliferation and viability at day 7 (A and B, respectively) or PDGF‐induced migration (C) was determined as described in Section 4. For migration assays (C), cells incubated with the vehicle in the absence of PDGF were included for comparative purposes. Data are presented as mean ± SEM from three independent experiments with values from vehicle‐treated cells arbitrarily assigned as 100% (A‐B) or 1 (C). Statistical analysis was performed using a one‐way ANOVA with Dunnett (2‐sided) post‐hoc test (*p* < 0.001).

In light of increase in plaque smooth muscle cell content in vivo (Figure 3), indicative of plaque stabilization, effect of CM on proliferation and migration of human aortic smooth muscle cells (HASMC) in response to key factor platelet‐derived growth factor BB (called PDGF hereafter)^[^ ^14^ ^]^ was determined. Linear regression analysis showed non‐significant reduction in proliferation with CM from Lab4 or CUL66 over a 7‐day period (data not shown). At day 7, CM from both Lab4 and CUL66 produced significant reduction in proliferation (*p* < 0.001 in both cases; **Figure** **10**A). Although treatment with CUL66 CM, but not Lab4 CM, showed significant reduction in cell viability (*p* < 0.001; Figure 10B), this reduction was less extensive than proliferation (Figure 10A). Finally, PDGF‐induced migration of HASMC was significantly attenuated by Lab4 CM (*p* < 0.001) whereas CUL66 CM had no significant effect (Figure 10C)

## 3 Discussion

Previous studies have shown beneficial effects of Lab4P in mice fed HFD and in a randomized controlled trial of overweight and obese adults.^[^ ^5^, ^6^ ^]^ However, the effects of Lab4P on atherosclerosis have not been determined and were investigated here in vivo and in vitro. Lab4P produced many anti‐atherogenic actions on plasma lipid profile, plaque burden, inflammation and a marker of stability, liver expression of atherosclerosis‐associated genes, immune cell profile in the bone marrow, macrophage foam cell formation and associated changes in gene expression and several others.

For weight parameters, the only significant change by Lab4P was an increase in spleen weight (Table 1). Increased spleen size is associated with reduced atherosclerosis and plaque development in LDLr^−/−^ mice^[^ ^15^ ^]^ whereas in ApoE^−/−^ mice fed an atherogenic diet, splenectomy accelerated atherosclerosis.^[^ ^16^ ^]^ The underlying mechanisms are not fully understood though spleen has a protective B cell response that is induced in atherosclerosis via inflammation leading to production of athero‐protective antibodies.^[^ ^17^ ^]^

For lipid profile, Lab4P produced a significant increase in HDL and a reduction in LDL/VLDL associated with favorable decrease in LDL/VLDL:HDL ratio (Table 1). Beneficial effects of probiotics on plasma cholesterol profile have been observed in other studies^[^ ^2^, ^5^ ^]^ and it is interesting that the liver expression of the *Apob* gene (protein part of atherogenic lipoproteins) was significantly decreased by Lab4P (Figure 4). In addition, CM from Lab4 or CUL66 increased macrophage cholesterol efflux associated with increased expression of genes for ABCA1/ABCG1 transporters and transcriptional activators LXR‐α/β (Figures 8 and 9), thereby suggesting reverse cholesterol transport and subsequent biliary secretion as one key potential mechanism. It should, however, be noted that not all changes in lipid profiles by Lab4P were beneficial as significantly increased levels of plasma TG (Table 1) were seen though previous studies with probiotics have indicated mixed results.^[^ ^2^ ^]^ The precise mechanisms underlying the increase in plasma TG levels remains to be determined. From the genes implicated in the control of TG homeostasis^[^ ^18^, ^19^ ^]^ Lab4P had no significant effect on the liver expression of *Lpl*, *Nr1h3* and *Ppard* genes but significantly decreases the expression of *pparg* and produces a trend towards increase in the expression of *Ppara* (Table S4, Supporting Information). Future studies should therefore investigate the impact of such changes in gene expression on plasma TG levels. As Lab4P decreases atherosclerosis development, it is likely that its actions on plasma HDL and LDL/VLDL levels along with plaque inflammation have a more dominant effect than the detrimental changes in plasma TG levels. Indeed, despite increasing plasma TG levels, Lab4P decreases the lipid content in the liver (Figure S1, Supporting Information).

Lab4P decreased plaque burden together with lipid and macrophage content (Figures 1 and 2). Decrease in plaque lipid content correlates well with changes in plasma LDL/VLDL and HDL levels but also with reduced macrophage uptake of oxLDL, associated with decreased expression of genes encoding scavenger receptors, and macropinocytosis together with increased cholesterol efflux and the expression of four key genes implicated in this process (Figures 8 and 9). The probiotic CM also produced an increase in macrophage phagocytosis (Figure 8F), which could potentially contribute to foam cell formation though this is also involved in the removal of apoptotic cells and debris from plaques.^[^ ^20^ ^]^ The decrease in plaque macrophage content also correlates with reduced chemokine‐driven monocytic migration and proliferation of monocytes and macrophages (Figures 6 and 7). Indeed, reduced macrophage proliferation has been associated with suppression of early atherosclerosis in LDLr^−/−^ mice whereas increased proliferation of monocytes and macrophages in ApoE^−/−^ mice accelerated disease development.^[^ ^21^ ^]^

The Lab4P‐mediated attenuation of plaque inflammation extended potentially to systemic inflammation as several genes whose liver expression was attenuated by the probiotics play critical roles in the control of immune and inflammatory responses (Figure 4), and this correlated with decreased frequency of macrophages and T cells in the bone marrow (Figure 5). The reduced expression of some of the genes is also consistent with decreased MCP‐1‐driven monocytic migration given that Ccr2 is the receptor for MCP‐1, *Icam* and *Sele* genes code for adhesion molecules ICAM1 and E‐Selectin, respectively, and IFN‐γ and TNF‐α are two key pro‐atherogenic cytokines.^[^ ^3^ ^]^ Interestingly, the liver expression of *Itgb2* that codes for integrin subunit β2, which is involved in the recognition of cell adhesion molecules on the activated endothelium,^[^ ^22^ ^]^ was also decreased by Lab4P (Figure 4).

Lab4P increased the plaque αSMA‐positive cell content (Figure 3) and in vitro assays showed that probiotic CM attenuated the proliferation and PDGF‐induced migration of HASMC (Figure 10). Proliferation of vascular smooth muscle cells (VSMC) plays an important role in atherogenesis where whilst controlled proliferation can be beneficial, dysregulated proliferation contributes to plaque formation.^[^ ^23^ ^]^ It is therefore likely that probiotic treatment may also have a beneficial effect on dysregulated proliferation. Other mechanisms are also likely; for example, key roles for matrix metallopeptidases (MMPs) in degradation of plaque stabilizing ECM produced by VSMC is well established^[^ ^24^ ^]^ and the liver expression of *Mmp1a* and *Mmp3*, which code for MMP‐1 and ‐3, respectively, was decreased by Lab4P (Figure 4). In addition, *Ifng* and *Il2* genes, whose liver expression was decreased by Lab4P (Figure 4), code for pro‐inflammatory cytokines IFN‐γ and IL‐2, respectively, that also impact VSMC function.^[^ ^24^ ^]^ Both IFN‐γ and IL‐2 induce the release of MMPs, thereby promoting the degradation of the extracellular matrix (ECM)^[^ ^24^ ^]^ and IFN‐γ also promotes apoptotic cell death of VSMC in atherosclerosis.^[^ ^25^ ^]^

LDLr^−/−^ mice were used in this study because they demonstrate more similarity to atherosclerosis development in humans compared to the ApoE^−/‐^ model.^[^ ^26^, ^27^ ^]^ ApoE also has a marked impact on inflammation, hematopoietic stem cell proliferation, monocytosis and monocyte accumulation in plaques so mice deficient in this apolipoprotein will potentially be compromised in such functions.^[^ ^26^, ^27^ ^]^ Nevertheless, future research should compare the action of Lab4P in both model systems for comparative purposes given that many previous studies on probiotics have used ApoE^−/−^ mice.^[^ ^2^ ^]^ The current study was restricted to the use of a single dose of Lab4P on atherosclerosis development at a single time point in male LDLr^−/−^ mice. Future research should therefore seek to investigate the effect of several doses of Lab4P on atherosclerosis development at more than one time point (e.g., early and late lesions) in both genders. The downstream analysis of Lab4P actions should also be extended to markers associated with HFD‐mediated changes in intestinal permeability and subsequent chronic inflammation (e.g., plasma levels of lipopolysaccharide and trimethylamine *N*‐oxide, translocation of fluorescein isothiocynate‐dextran from the gut into circulation, etc.) together with markers of endothelial dysfunction, an early step in atherosclerosis (e.g., expression of adhesion molecules). In addition, the current study involved feeding animals a HFD so further research should investigate the effect of Lab4P on atherosclerosis development in animals fed a chow diet. Finally, the effect of Lab4P on regression of existing atherosclerotic plaques should be analysed.

In conclusion, this is the first study that demonstrates an anti‐atherogenic action of Lab4P in vitro and in vivo and provides novel mechanistic insights that supports further assessments in human trials. In addition, this study adds to our previous demonstration of Lab4P‐mediated reduction in plasma total cholesterol levels and diet‐induced weight gain in an acute study in mice fed a HFD^[^ ^5^ ^]^ and a reduction of body weight and improvement of well‐being in a randomized control trial of overweight and obese adults.^[^ ^6^ ^]^

## 4 Experimental Section

### Materials

All reagents were from Sigma‐Aldrich (Poole, UK) or ThermoFisher Scientific (Paisley, UK) unless otherwise stated.

### Animal Experiments

Studies were approved by the Cardiff University Institutional Ethics Review Committee and the United Kingdom Home Office, and experiments were performed in accordance with the Guide for the Care and Use of Laboratory Animals (NIH Publication No. 85–23, revised 1996; Experimental licence 30/3365). LDLr^−/−^ mice homozygous for the *LDLrtm1Her* mutation and backcrossed to the C57BL/6J strain were expanded locally in a pathogen‐free environment.

Male LDLr^−/‐^ mice (8‐weeks‐old) were randomly assigned to two groups (n = 15) and fed HFD [21% w/w pork lard and 0.15% w/w cholesterol] (Special Diets Services, Witham, UK) or HFD supplemented with Lab4P [5 × 10^8^ colony forming units (CFU)/mouse/day; 100 billion CFU/day equivalent human dose)^[^ ^6^ ^]^ for 12 weeks housed in a pathogen‐free scantainers ventilated cages in a light and temperature‐controlled facility (12‐h light/dark cycle, 22 °C). Mouse body weights were measured every 2 days together with approximate food consumed per mouse per day. The mice were sacrificed at the end of the study using carbon dioxide anesthesia and death confirmed by an absence of a pulse.

Whole blood from cardiac puncture was collected into tubes containing heparin (5000 U mL^‐1^) and the heart flushed with phosphate buffered saline (PBS). Plasma was obtained by centrifugation for 5 min at 12,000 x *g* and stored at ‐80 °C. The fat pads and organs were weighed, snap frozen in liquid nitrogen and stored at ‐20 °C. Rear legs were removed for subsequent analysis of cell populations in the bone marrow.

### Plasma Lipid Profile

These were determined using the Cholesterol Assay Kit ‐HDL and LDL/VLDL (ab65390) and Triglyceride (TG) Assay Kit (ab65336) according to the manufacturer's instructions (Abcam, Cambridge, UK). The Cholesterol Assay Kit allowed separation via precipitation (using the buffer provided in the kit) of HDL and LDL/VLDL and then determination of cholesterol levels in these particles.

### Analysis of Aortic Root Sections by H & E and ORO Staining, and IHC

The heart with the aortic root and the liver were stored in cryomolds with optimum cutting temperature formulation at ‐80 °C. Serial sections (10 µm) of the aortic roots, containing all three valves, and the liver were then taken at ‐20 °C, collected onto poly‐l‐lysine coated slides, and air‐dried for 1 h before storage at ‐80 °C.

For the aortic root, sections were rinsed in distilled water for 5 min and stained in 5% w/v Gill's hematoxylin [0.1% w/v hematoxylin, 0.02% v/v sodium iodate and 2% v/v glacial acetic acid] (Abcam, Cambridge, UK) for 5 min. The slides were then rinsed in water, counterstained in eosin for 10 min and dehydrated by passing through 80%, 95%, and 100% ethanol for 5 min each. The slides were then incubated in xylene for 5 min. For ORO staining, sections were fixed in 4% w/v paraformaldehyde for 15 min, washed three times in distilled water and counterstained in Gill's hematoxylin for 3 min. After washing with water, slides were placed in absolute propylene glycol before staining with ORO (Abcam, Cambridge, UK) for 15 min at 60 °C. The slides were then incubated in 85% propylene glycol for 5 min and washed three times in distilled water. For the liver, the sections were stained with ORO for 18 min at room temperature, counterstained with Gill's hematoxylin for 2 min and washed with water. In all cases, the sections were mounted using aqueous mountant, sealed with a coverslip and images captured using a Leica DMRB brightfield microscope with ProgRes CapturePro 2.8.8 software [X40 magnification (X4 objective) for aortic root and X10 or X20 magnification (X2.5 objective) for the liver].

Immunofluorescent staining was performed using cell surface marker antibodies for macrophages (rat anti‐mouse MOMA‐2, 1 in 100 dilution of 0.5 mg mL^‐1^ stock), smooth muscle cells (rabbit anti‐mouse α‐smooth muscle actin, 1 in 100 dilution of 0.2 mg mL^‐1^ stock) and T cells (rabbit anti‐mouse CD3, 1 in 100 dilution of 0.2 mg mL^‐1^ stock) or appropriate isotype control antibodies (all from Abcam, Cambridge, UK). Sections were fixed in acetone for 15 min, washed twice in PBS and non‐specific interactions blocked by incubation with 5% v/v serum (dependent on species of secondary antibody) in 5% w/v BSA in PBS for 30 min. Following incubation overnight at 4 °C with primary antibody or appropriate isotype control (1/100 dilution), slides were washed twice in PBS and the corresponding secondary antibodies (Chicken anti‐rat IgG AF‐594 or Donkey anti‐rabbit IgG AF‐488; 1 in 500 dilution of 2 mg mL^‐1^ stock) applied for 1 h in the dark. Slides were then washed twice with PBS, counterstained for 20 min in 0.3% w/v Sudan Black, washed three times in PBS and mounted in Fluoroshield with DAPI nuclear stain. Images were captured using an Olympus BX61 microscope with analySIS v3.2 software at X40 magnification (X4/0.13 objective). DAPI, TRITC and FITC filters were applied for imaging of DAPI, AF‐594 and AF‐488 fluorophores, respectively.

All images were captured using consistent exposure, intensity and contrast settings for comparable downstream analyses. In all cases, regions of staining were quantified in a blinded manner using the ImageJ software.

### Immunophenotyping of Bone Marrow Cell Populations

Immunophenotyping of bone marrow cell populations was performed using marker antibodies and reagents (Table S1 and S2, Supporting Information) as previously described.^[^ ^12^ ^]^ Thus, 10 or 8 million cells, respectively, were used to analyze the signaling lymphocytic activation molecule (SLAM) and progenitor cell populations whereas 1 million cells were employed for lineage cell populations. Samples were analyzed on a BD LSR Fortessa 4 lasers flow cytometer and data analysis performed using FlowJo v.10 software.

### Preparation of Probiotic CM

Lyophilised probiotics were cultured in de Man, Rogosa and Sharpe Agar broth (0.1 mg mL^‐1^) for 18 h at 37 °C under low oxygen and non‐agitating conditions (80% N_2_, 10% CO_2_, 10% O_2_, v/v). Bacteria were then pelleted by centrifugation (10 min, 1000 x *g*), washed in 1X PBS and resuspended in the same volume of cell culture media (RPMI‐1640 or DMEM depending on the cells). Suspensions were incubated for a further 5 h at 37 °C as above. The bacteria were pelleted by centrifugation as above, CM was collected, pH adjusted to 7.4 using 0.5 M NaOH, treated with penicillin‐streptomycin (both 10000 U mL^‐1^) and filter sterilized using 0.22 µm pore filters. Protein quantification was carried out on each batch of CM using the Pierce Coomassie Plus Assay Kit. Once thawed, CM was not subjected to any further freeze/thaw cycles.

### Cell Culture

THP‐1 monocytes were cultured and differentiated into macrophages as previously described.^[^ ^10^, ^28^ ^]^ Primary HASMCs were cultured in smooth muscle cell growth medium according to the manufacturer's instructions (Sigma‐Aldrich). HMDMs were isolated from buffy coats from the National Blood Service Wales as previously described^[^ ^10^, ^28^ ^]^ except that differentiation was performed in the presence of 20 ng mL^‐1^ human macrophage colony stimulating factor. Informed consent was granted from each donor for their use and all experiments were approved by Cardiff University.

### In Vitro Assays

Cell viability, proliferation, monocyte chemotactic protein (MCP)‐1‐driven monocytic migration, uptake of Dil‐labeled oxidized LDL (oxLDL), macropinocytosis using Lucifer Yellow and radioactive‐based cholesterol efflux from foam cells were performed as previously described.^[^ ^10^, ^28^ ^]^ The levels of ROS in cells in vitro were assessed using a 2’7’‐dichlorofluorescin diacetate (DCFDA) Cellular ROS Detection Assay Kit (ab113851) according to the manufacturer's instructions (Abcam). TBHP (50 µM) was used as a positive control for ROS production in cells. BrdU incorporation and phagocytosis was performed using the BrdU labeling and detection kit III and Vybrant Phagocytosis Assay Kit, respectively, as described by the manufacturer (Sigma‐Aldrich and ThermoFisher Scientific, respectively). HASMC migration was investigated using a modified Boyden chamber with Matrigel‐coated membrane and human PDGF as a chemoattractant.

### Analysis of Gene Expression

Preparation of RNA, conversion to cDNA and either standard real‐time quantitative PCR (RT‐qPCR) using primers shown in Table S3, Supporting Information or Atherosclerosis RT^2^ Profiler PCR Arrays (Qiagen, Manchester, UK) was performed as previously described.^[^ ^10^, ^12^, ^28^ ^]^ Gene transcript levels were calculated using the comparative Ct method.

### Statistical Analysis of the Data

Q‐Q plots, Shapiro‐Wilk test and histograms were used to assess normality of data (values outside of 2 standard deviations of the mean were removed as outliers prior to statistical analysis). For two groups, either a Student's *t*‐test or Mann‐Whitney *U* test was performed. For more than two groups, a one‐way analysis of variance (ANOVA) was performed with homogeneity of variance analyzed using Levene's statistical test and post‐hoc tests selected with the Dunnett 2‐sided post‐hoc test performed where equal variances were present or Dunnett T3 in the case of unequal variances. Analysis was performed using IBM SPSS Statistics 23 software unless otherwise stated (significance defined as *p* ≤ 0.05).

## Conflict of Interest

This study was supported in part by Cultech Ltd, Port Talbot, UK. D.R.M. and S.F.P. are employees of Cultech Ltd. V.L.M. Ph.D. was funded by a Knowledge Economy Skills Scholarship with Cultech Ltd.

## Author Contributions

V.L.M., Y.H.C., J.O.W., N.P.R., S.F.P., T.R.H., D.R.M., and D.P.R. were responsible for the design of the experiments. Experiments were performed by V.L.M., R.A., and A.A., and data analysis was carried out by V.L.M., Y.H.C., R.A., and A.A. V.L.M., Y.H.C., R.A., and A.A. prepared the figures and V.L.M. and D.P.R. wrote the manuscript. All the authors contributed to the review of the manuscript.

## Supporting information

Supporting Information

## Data Availability

The data that support the findings of this study are available from the corresponding author upon reasonable request.
