# Supplementary material for: The Lab4P Consortium of Probiotics Attenuates Atherosclerosis in LDL Receptor Deficient Mice Fed a High Fat Diet and Causes Plaque Stabilization by Inhibiting Inflammation and Several Pro‐Atherogenic Processes
Source: Mol Nutr Food Res. 2021 Jul 19;65(17):2100214. doi: 10.1002/mnfr.202100214 (PMC9373067; doi:10.1002/mnfr.202100214)
Supplement: Supplementary file 1 — Supporting Information [file MNFR-65-0-s001.pdf]

A consortium of probiotics, Lab4P, attenuates atherosclerosis in LDL receptor deficient mice fed a high fat diet and causes plaque stabilization by inhibiting inflammation and several other pro-atherogenic processes

Victoria L. O'Morain[1], Yee-Hung Chan[1], Jessica O. Williams[1], Reem Alotibi[1], Alaa Alahmadi[1], Neil P. Rodrigues[2], Sue F. Plummer[3], Timothy R. Hughes[4], Daryn R. Michael[3] and Dipak P. Ramji[1]*

[1]Cardiff School of Biosciences, Cardiff University, Sir Martin Evans Building, Museum Avenue, Cardiff CF10 3AX, UK.
[2]Systems Immunity Research Institute, School of Medicine, Cardiff University, Cardiff CF14 4XN, UK.
[3]European Cancer Stem Cell Research Institute, Cardiff School of Biosciences, Cardiff University, Hadyn Ellis Building, Maindy Road, Cardiff CF24 4HQ, UK.
[4]Cultech Limited, Unit 2 Christchurch Road, Baglan Industrial Park, Port Talbot, SA12 7BZ, UK.

***Corresponding author:** Professor Dipak P. Ramji, Cardiff School of Biosciences, Cardiff University, Sir Martin Evans Building, Museum Avenue, Cardiff CF10 3AX, UK. Tel: 0044 (0)29 20876753; Fax: 0044 (0)29 20874116; Email: Ramji@Cardiff.ac.uk

Running title: Anti-atherogenic actions of Lab4P

Supplementary Table 1. Details of reagents used for flow cytometry

Reagent	Fluorochrome where applicable	Supplier	Clone	Catalogue number
SLAM CLASS				
Ly-6A/E (Sca-1)	PE	BioLegend	D7	108107
CD48	FITC	BioLegend	HM48-1	103403
CD150	PE/Cy7	BioLegend	TC15-12F12.2	115913
CD117 (c-Kit)	APC	BioLegend	2B8	105811
Lineage cocktail				
2% PBS-FCS				
PROGENITOR				
Ly-6A/E (Sca-1)	APC/Cy7	BioLegend	D7	108125
CD127 (IL-7Rα)	PE	BioLegend	SB/199	121111
CD117 (c-Kit)	APC	BioLegend	2B8	105811
Lineage cocktail				
2% PBS-FCS				
LINEAGE				
Ly-6G/Ly-6C (Gr-1)	PE/Cy7	BioLegend	RB6-8C5	108415
CD11b (Mac-1)	PE	BioLegend	M1/70	101207
CD45R/B220	APC	BioLegend	RA3-6B2	103212
CD3	FITC	BioLegend	17A2	100203
TER-119	APC/Cy7	BioLegend	TER-119	116223
2% PBS-FCS				
LINEAGE COCKTAIL				
Biotin CD3		BioLegend	17A2	100244
Biotin CD4		BioLegend	GK1.5	100404
Biotin CD8a		BioLegend	53-6.7	100703
Biotin Ly-6G/Ly-6C (Gr-1)		BioLegend	RB6-8C5	108404
Biotin CD11b		BioLegend	M1/70	101204
Biotin CD45R/B220		BioLegend	RA3-6B2	103204
Biotin TER-119		BioLegend	TER-119	116204

Supplementary Table 2. Markers used in immunophenotyping of bone marrow cell populations

Class	Cell type	Identifier
SLAM	Lineage –<br>LSK<br>HSC<br>MPP<br>HPCI<br>HPCII	 Lin^- $Sca\text{-}1^+$ $c\text{-}Kit^+$<br>$CD150^+$ $CD48^-$<br>$CD150^-$ $CD48^-$<br>$CD150^-$ $CD48^+$<br>$CD150^+$ $CD48^+$
Progenitor	Lineage –<br>CLP	 $CD127^+$
Lineage	Lineage +<br>Granulocyte<br>MDSC<br>Macrophages<br>B-Cell<br>T-Cell	 $GR1^+$ $Mac1^-$<br>$GR1^+$ $Mac1^+$<br>$GR1^-$ $Mac1^+$<br>$B220^+$<br>$CD3^+$

Abbreviations: SLAM, signalling lymphocyte activation molecule; HSC, hematopoietic stem cell; MPP, multipotent progenitors; HPC, hematopoietic progenitor cell; CLP, common lymphoid progenitor; MDSC, myeloid-derived suppressor cells.

Supplementary Table 3. Sequences of primers used for RT-qPCR

Target Gene	Forward Primer Sequence (5' to 3')	Reverse Primer Sequence (5' to 3')
GAPDH	CTTTTGCGTCGCCAGCCGAG	GCCCAATACGACCAAATCCGTTGACT
CD36	AGCCATTTTAAAGATAGCTTTCC	AAGCTCTGGTTCTTATTCACA
SR-A	GTCCAATAGGTCCTCCGGGT	CCCACCGACCAGTCGAAC
LPL	GAGATTTCTCTGTATGGCACC	CTGCAAATGAGACACTTTCTC
ABCA1	AGTGGAAACAGTTAATGACCAG	GCAGCTGACATGTTTGTCTTC
ABCG1	GGTGGACGAAGAAAGGATACAAGACC	ATGCCCGTCTCCCTGTATCCA
LXRα	CCTTCAGAACCCACAGAGATCC	ACGCTGCATAGCTCGTTCC
LXRβ	GCTAACAGCGGCTCAAGAACT	GGAGCGTTTGTTGCACTGC
ApoE	CAGGAGCCGACTGGCCAATC	ACCTTGGCCTGGCATCCTG

Abbreviations: GAPDH, glyceraldehyde 3-phosphate dehydrogenase; SR-A, scavenger receptor-A; LPL, lipoprotein lipase; LXR, liver X receptor; ABCA1, ATP-binding cassette transporter A1; ABCG1, ATP-binding cassette transporter G1; ApoE, apolipoprotein E.

Supplementary Table 4. The effect of Lab4P on the liver expression of atherosclerosis-associated genes

Gene	GenBank ID	Fold-change	*P* value	Change
Abca1	NM_013454	1.27	0.129	Increase
Ace	NM_009598	1.19	0.403	Decrease
Apoa1	NM_009692	1.06	0.756	Increase
Apob	NM_009693	1.18	0.048	Decrease
Apoe	NM_009696	1.05	0.372	Increase
Bax	NM_007527	1.41	0.073	Decrease*
Bcl2	NM_009741	1.23	0.275	Increase
Bcl2a1a	NM_009742	1.19	0.704	Decrease
Bcl2l1	NM_009743	1.47	0.074	Decrease*
Bid	NM_007544	1.18	0.427	Decrease
Birc3	NM_007464	1.03	0.572	Increase
Ccl2	NM_011333	2.08	0.111	Decrease
Ccl5	NM_013653	1.79	0.179	Decrease
Ccr1	NM_009912	1.70	0.003	Decrease
Ccr2	NM_009915	1.63	0.006	Decrease
Cd44	NM_009851	1.93	0.178	Decrease
Cdh5	NM_009868	1.08	0.980	Decrease
Cflar	NM_009805	1.23	0.017	Decrease
Col3a1	NM_009930	2.38	0.253	Decrease
Csf2	NM_009969	1.79	0.317	Decrease
Ctgf	NM_010217	1.25	0.814	Decrease
Cxcl1	NM_008176	3.09	0.087	Decrease*
Eln	NM_007925	1.07	0.684	Increase
Eng	NM_007932	1.10	0.311	Increase
Fabp3	NM_010174	1.21	0.241	Increase
Fas	NM_007987	1.19	0.315	Decrease
Fga	NM_010196	1.12	0.542	Decrease
Fgb	NM_181849	1.30	0.211	Increase

Gene	GenBank ID	Fold-change	*P* value	Change
Fgf2	NM_008006	1.11	0.807	Decrease
Fn1	NM_010233	1.30	0.124	Increase
Hbegf	NM_010415	1.13	0.024	Decrease
Icam1	NM_010493	1.34	0.025	Decrease
Ifng	NM_008337	2.40	<0.001	Decrease
Il1a	NM_010554	1.47	0.110	Decrease
Il1b	NM_008361	1.03	0.477	Increase
Il1r1	NM_008362	1.17	0.007	Decrease
Il1r2	NM_010555	1.30	0.815	Decrease
Il2	NM_008366	1.76	0.048	Decrease
Il3	NM_010556	1.12	0.154	Increase
Il4	NM_021283	1.21	0.183	Decrease
Il5	NM_010558	1.29	0.435	Decrease
Itga2	NM_008396	1.72	<0.001	Decrease
Itga5	NM_010577	1.67	0.620	Decrease
Itgax	NM_021334	1.23	0.246	Decrease
Itgb2	NM_008404	1.32	0.001	Decrease
Kdr	NM_010612	1.05	0.462	Increase
Klf2	NM_008452	1.29	0.695	Increase
Lama1	NM_008480	1.28	0.171	Decrease
Ldlr	NM_010700	1.00	0.713	No change
Lif	NM_008501	1.03	0.540	Increase
Lpl	NM_008509	1.21	0.161	Decrease
Lypla1	NM_008866	2.52	0.014	Decrease
Mmp1a	NM_032006	1.80	<0.001	Decrease
Mmp3	NM_010809	1.50	0.018	Decrease
Msr1	NM_031195	1.40	0.351	Decrease
Nfkb1	NM_008689	1.09	0.310	Increase
Npy	NM_023456	1.86	0.282	Decrease

Gene	GenBank ID	Fold-change	*P* value	Change
Nr1h3	NM_013839	1.05	0.395	Increase
Pdgfa	NM_008808	1.31	0.019	Increase
Pdgfb	NM_011057	1.07	0.678	Decrease
Pdgfrb	NM_008809	1.06	0.900	Increase
Plin2	NM_007408	1.12	0.187	Decrease
Ppara	NM_011144	1.09	0.074	Increase*
Ppard	NM_011145	1.12	0.153	Increase
Pparg	NM_011146	1.40	0.046	Decrease
Ptgs1	NM_008969	1.04	0.455	Increase
Rxra	NM_011305	1.32	0.239	Increase
Sele	NM_011345	1.56	0.005	Decrease
Sell	NM_011346	1.42	0.414	Decrease
Selp	NM_011347	1.17	0.171	Decrease
Selplg	NM_009151	1.12	0.554	Decrease
Serpinb2	NM_011111	1.12	0.553	Decrease
Serpine1	NM_008871	2.27	0.001	Decrease
Sod1	NM_011434	1.02	0.639	Increase
Spp1	NM_009263	1.00	0.515	No change
Tgfb1	NM_011577	1.09	0.266	Increase
Tgfb2	NM_009367	1.17	0.818	Decrease
Thbs4	NM_011582	1.09	0.385	Increase
Tnc	NM_011607	1.16	0.349	Decrease
Tnf	NM_013693	1.28	0.035	Decrease
Tnfaip3	NM_009397	2.26	0.098	Decrease*
Vcam1	NM_011693	1.37	0.082	Decrease*
Vegfa	NM_009505	1.51	0.126	Decrease
Vwf	NM_011708	1.13	0.336	Increase

The table shows gene, GenBank reference, fold-change in expression, *p* value and increase/decrease/no change in gene expression. Statistical analysis was performed using an unpaired Student's t-test. * Trend towards significance where *p* values are between 0.05 and 0.100. *Abbreviations*: *Abca1*, ATP-binding cassette, subfamily A (ABC1), member 1; *Ace*,

angiotensin I converting enzyme (peptidyl-dipeptidase A) 1; *Actb*, β-actin; *Appa1*, apolipoprotein A-I; *Apob*, apolipoprotein B; *Apoe*, apolipoprotein E; *Bax*, BCL2-associated X protein; *Bcl2*, B cell leukemia/lymphoma 1; *Bcl2a1a* (Bfl-1, A1), B cell leukemia/lymphoma 2 related protein A1a; *Bcl2l1* (Bcl-XL), BCL2-like 1; *Bid*, BH3 interacting domain death agonist; *Birc3* (cIAP1, cIAP2), buculoviral IAP repeat-containing 3; *Ccl2* (MCP-1), chemokine (C-C motif) ligand 2; *Ccl5* (RANTES), chemokine (C-C motif) ligand 5; *Ccr1*, chemokine (C-C motif) receptor 1; *Cdh5*, cadherin 5; *Ccr2*, chemokine (C-C motif) receptor 2; *Cd44*, CD44 antigen; *Cflar* (Casper), CASP8 and FADD-like apoptosis regulator; *Col3a1*, collagen, type III, α1; *Cxcl1* (Gro1), chemokine (C-X-C motif) ligand 1; *Csf2* (GMCSF), colony stimulating factor 2 (granulocyte-macrophage); *Ctgf*, connective tissue growth factor; *Eln*, elastin; *Eng* (Evi-1), endoglin; *Fabp3*, fatty acid binding protein 3, muscle and heart; *Fas* (TNFRSF6), TNF receptor superfamily member 6; *Fga*, fibrinogen α chain; *Fgb*, fibrinogen β chain; *Fgf2* (bFGF), fibroblast growth factor 2; *Fn1*, fibronectin 1; *Hbegf* (Dtr), heparin-binding EGF-like growth factor; *Icam1*, intercellular adhesion molecule 1; *Ifng*, interferon-γ; *IL1a*, interleukin-1α; *IL1b*, interleukin-1β; *IL1r1*, interleukin 1 receptor, type I; *IL1r2*, interleukin 1 receptor, type II; *Il2*, interleukin-2; *Il3*, interleukin-3; *Il4*, interleukin-4; *Il5*, interleukin-5; *Itga2*, integrin α2; *Itga5*, integrin α 5 (fibronectin receptor α); *Itgax*, integrin α X; *Itgb2*, integrin β2; *Kdr* (VEGFR2), kinase insert domain protein receptor; *Klf2*, kruppel-like factor 2 (lung); *Lama1*, laminin α1; *Ldlr*, low density lipoprotein receptor; *Lif*, leukemia inhibitory factor; *Lpl*, lipoprotein lipase; *Lypla1*, lysophospholipase 1; *Mmp1a*, matrix metallopeptidase 1a (interstitial collagenase); *Mmp3*, matrix metallopeptidase 3; *Msr1*, macrophage scavenger receptor 1; *Nfkb1*, nuclear factor of kappa light polypeptide gene enhancer in B cells 1, p105; *Npy*, neuropeptide Y; *Nr1h3*, nuclear receptor subfamily 1, h group H, member 3; *Pdgfa*, platelet derived growth factor α; *Pdgfb*, platelet derived growth factor, B polypeptide; *Pdgfrb*, platelet derived growth factor receptor, beta polypeptide; *Plin2*, perlipin 2; *Ppara*, peroxisome proliferator activated receptor α; *Ppard*, peroxisome proliferator activated receptor δ; *Pparg*, peroxisome proliferator activated receptor γ; *Ptgs1* (COX1), prostaglandin-endoperoxide synthase 1; *Rxra*, retinoid X receptor α; *Sele*, selectin, endothelial cells; Sell (LECAM-1), selectin, lymphocyte; *Selp*, selectin, platelet; *Sod1*, superoxide dismutase 1; *Selplg* (P-Selectin), selectin, platelet (p-selectin) ligand; *Serpinb2* (PAI-2), serine (or cysteine) peptidase inhibitor, clade B, member 2; *Serpine1* (PAI-1), serine (or cysteine) peptidase inhibitor, clade B, member 1; *Spp1*, secreted phosphoprotein 1; *TGFb1*, transforming growth factor-β1; *TGFb2*, transforming growth factor-β1; *Thbs4*, thrombospondin 4; *Tnc*, tenascin C; *Tnf*, tumor necrosis factor; *Tnfaip3*, tumor necrosis factor, alpha-induced protein 3; *Vcam1*, vascular cell adhesion molecule 1; *Vegfa*, vascular endothelial growth factor A; *Vwf*, Von Willebrand factor

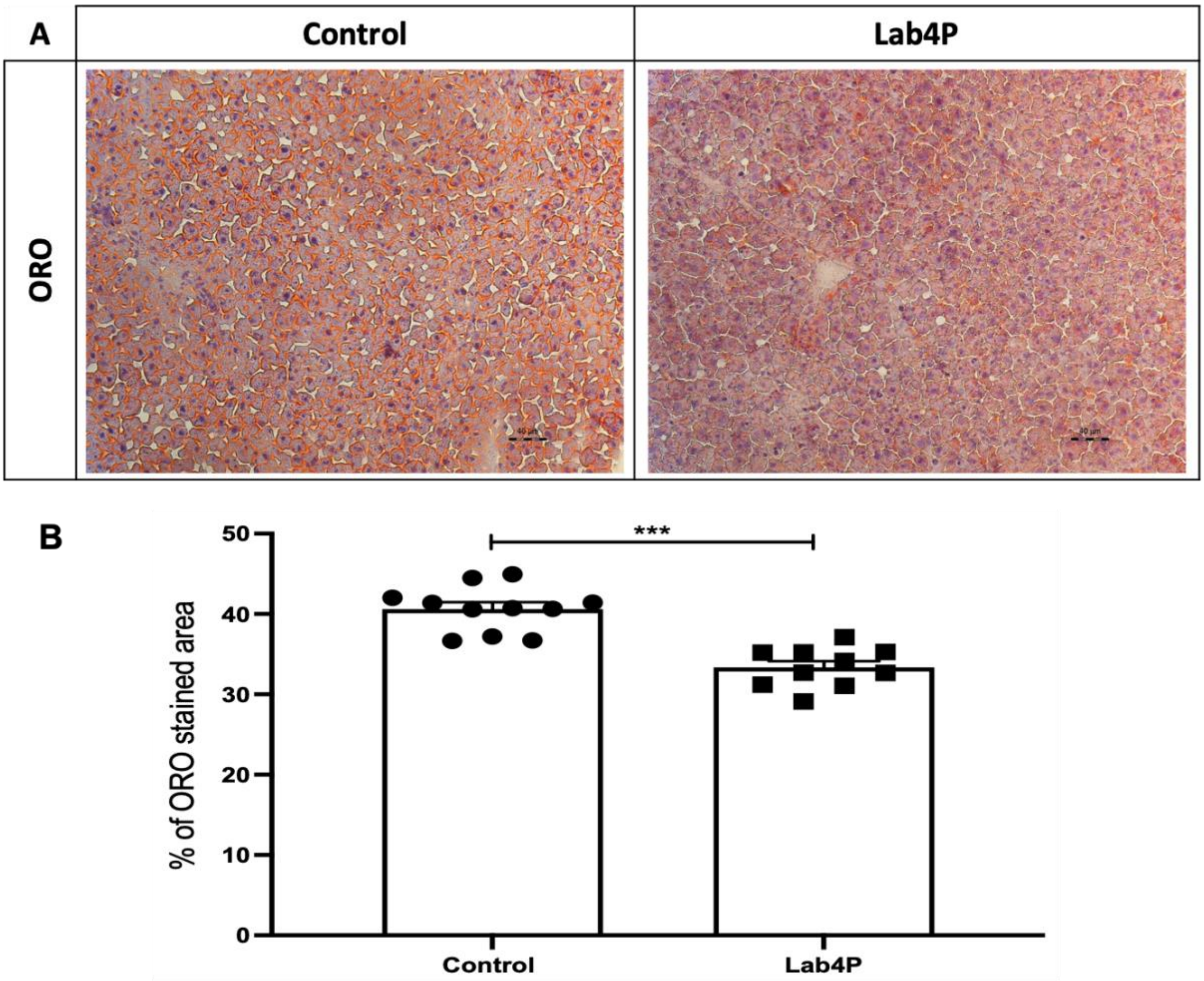

Supplementary Figure 1. Lab4P produces a decrease in liver lipid content in LDLr$^{-/-}$ mice fed HFD

Sections from the liver of mice fed HFD for 12 weeks (Control) or HFD supplemented with Lab4P (Lab4P) were stained with Oil Red O (ORO) and then counterstained with Gill's haematoxylin. **(A)** shows representative images from this staining (x20 magnification; scale bar of 40 μm). **(B)** shows the percentage of ORO stained area as determined using the Image J software. Data are mean ± SEM (n=11 for Control and 10 for Lab4P). Statistical analysis was carried out using an unpaired Student's t test (*, p≤0.001).

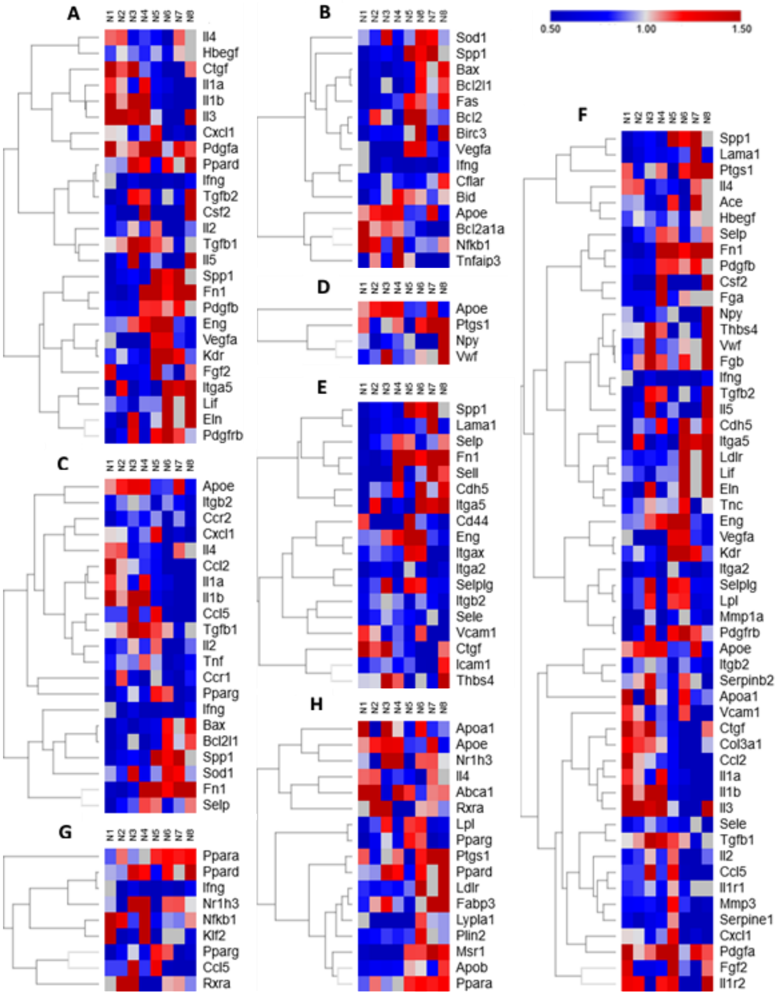

Supplementary Figure 2. Heatmaps showing changes in liver expression of atherosclerosis-associated genes following Lab4P supplementation.

The effect of Lab4P on atherosclerosis-associated gene expression was assessed in livers from LDLr$^{-/-}$ mice fed for 12 weeks with either a HFD (control group; n=8) or a HFD supplemented with Lab4P (n=8). Each heatmap consists of a family of genes with related functions: (A) Cell growth and proliferation; (B) Apoptosis; (C) Stress responses; (D) Blood coagulation and circulation; (E) Cell adhesion molecules; (F) Extracellular matrix molecules; (G) Transcriptional regulation; and (H) Lipid transport and metabolism. Box colour and intensity represents fold-change in gene expression as depicted by the scale. Clustering displayed on the left of each heatmap is according to similarity of expression pattern between genes in each related group. Fold-change in gene expression was determined using the ΔΔCT method. Cluster analysis was performed and heatmaps produced using Morpheus software. A list of full gene names is included in Supplementary Table 4.

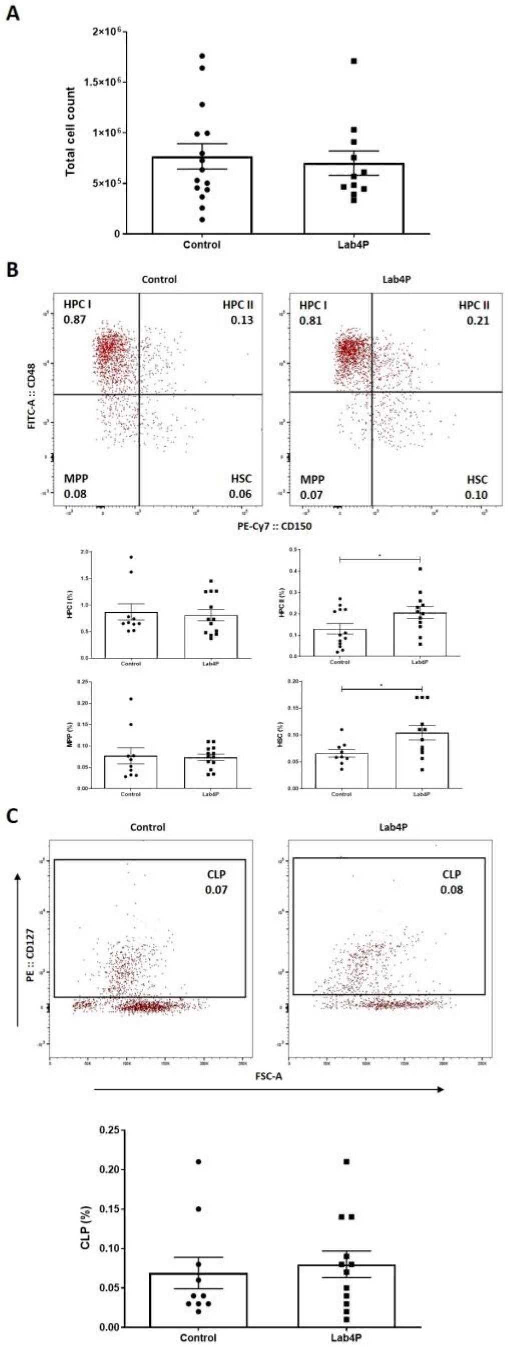

Supplementary Figure 3. The effect of Lab4P on total cell counts together with stem and progenitor cell populations in the bone marrow

Immunophenotyping of bone marrow cell populations from LDLr$^{-/-}$ mice was performed following 12 weeks feeding with either a HFD (Control; n=15) or a HFD supplemented with Lab4P (n=15). **(A)** The graph shows total cell counts from the Control and Lab4P groups. **(B)** Representative flow plots of stem cell populations in control and Lab4P groups are shown on top with gating indicating the position of populations representing HPC I (CD48^{+}CD150^{-}), HPC II (CD48^{+}CD150^{+}), MPP (CD48^{-}CD150^{-}) and HSC (CD48^{-}CD150^{+}) cells. Graphs below the flow plots show the frequency of HPC I, HPC II, MPP and HSC populations. **(C)** Representative flow plots showing CLP progenitor cell populations in control and Lab4P groups with graph showing the frequency of CLP population. Data are mean ± SEM with statistical analysis performed using an unpaired Student’s t-test (**p* <0.05).

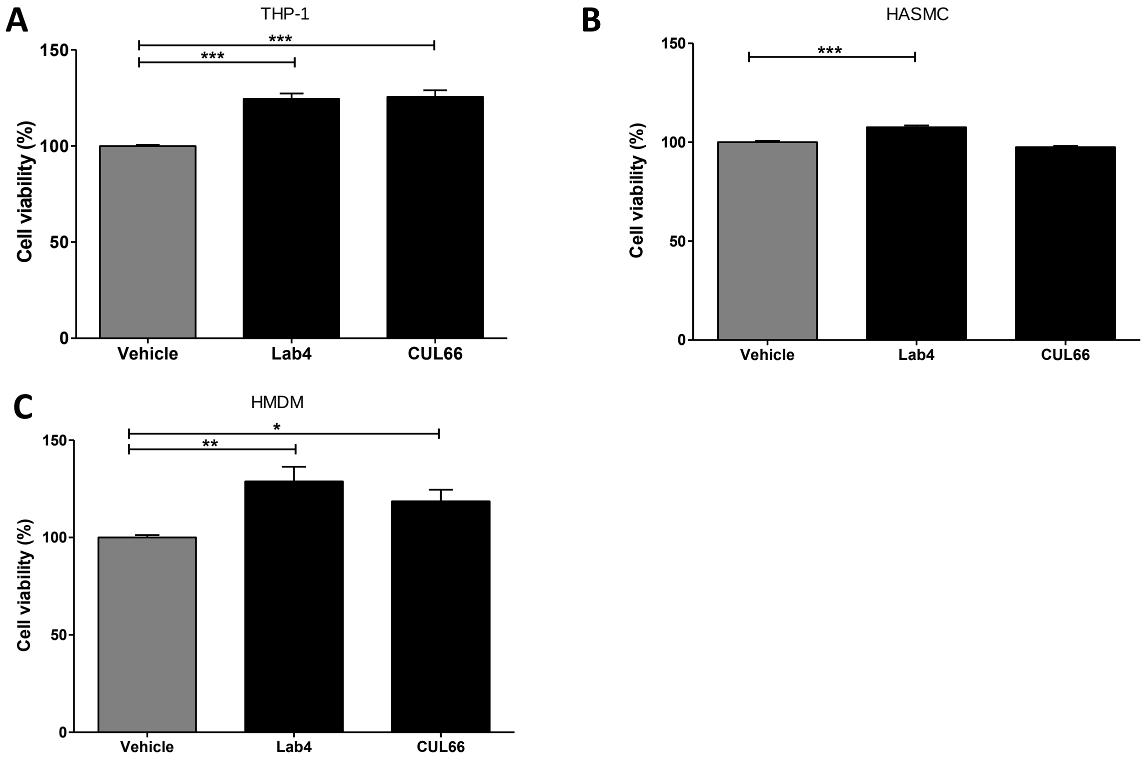

Supplementary Figure 4. Probiotic CM has no detrimental effect on the viability of cell culture model systems used in this study. **(A)** THP-1 macrophages, **(B)** HASMCs and **(C)** HMDMs were treated for 24 h with the vehicle control (Vehicle) or CM from Lab4 (8 µg/ml) or CUL66 (5 µg/ml) as indicated. Cell viability was assessed by following the release of the LDH enzyme. Cell viability was determined as a percentage relative to the vehicle control, which was arbitrarily assigned as 100%. Data are presented as mean ± SEM from three independent experiments. Statistical analysis was performed using a one-way ANOVA with Dunnett T3 post-hoc test where *p <0.05, **p <0.01 and *** p <0.001.

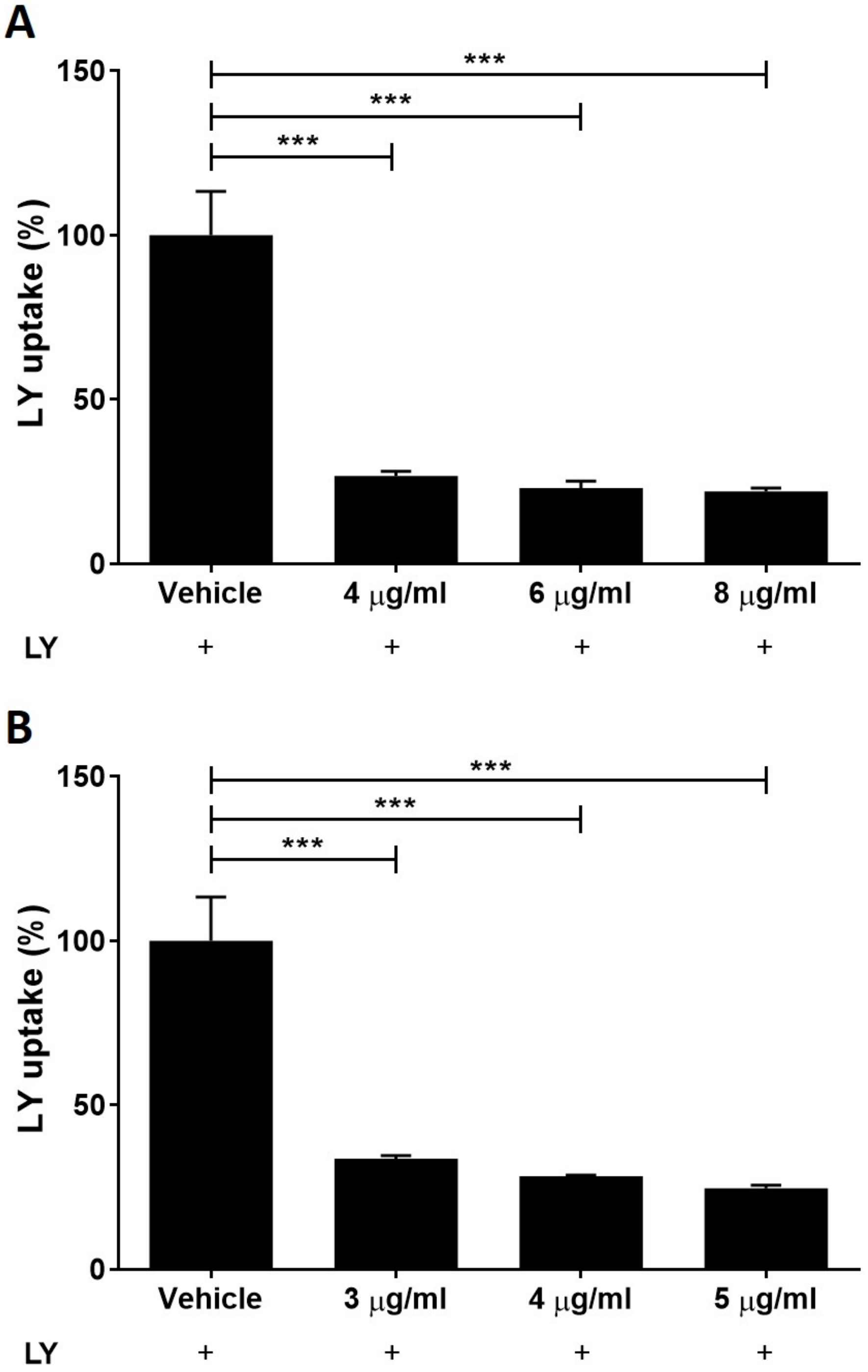

Supplementary Figure 5. Probiotic CM attenuates macropinocytosis in human macrophages at several different concentrations. Macropinocytosis was determined following incubation with LY and either vehicle or the indicated concentration of CM from **(A)** Lab4 or **(B)** CUL66 (the value from vehicle-treated cells was arbitrarily assigned as 100%). Data are presented as mean ± SEM from three independent experiments. Statistical analysis was performed using a one-way ANOVA with Dunnett 2-sided post-hoc test (*** p <0.001).

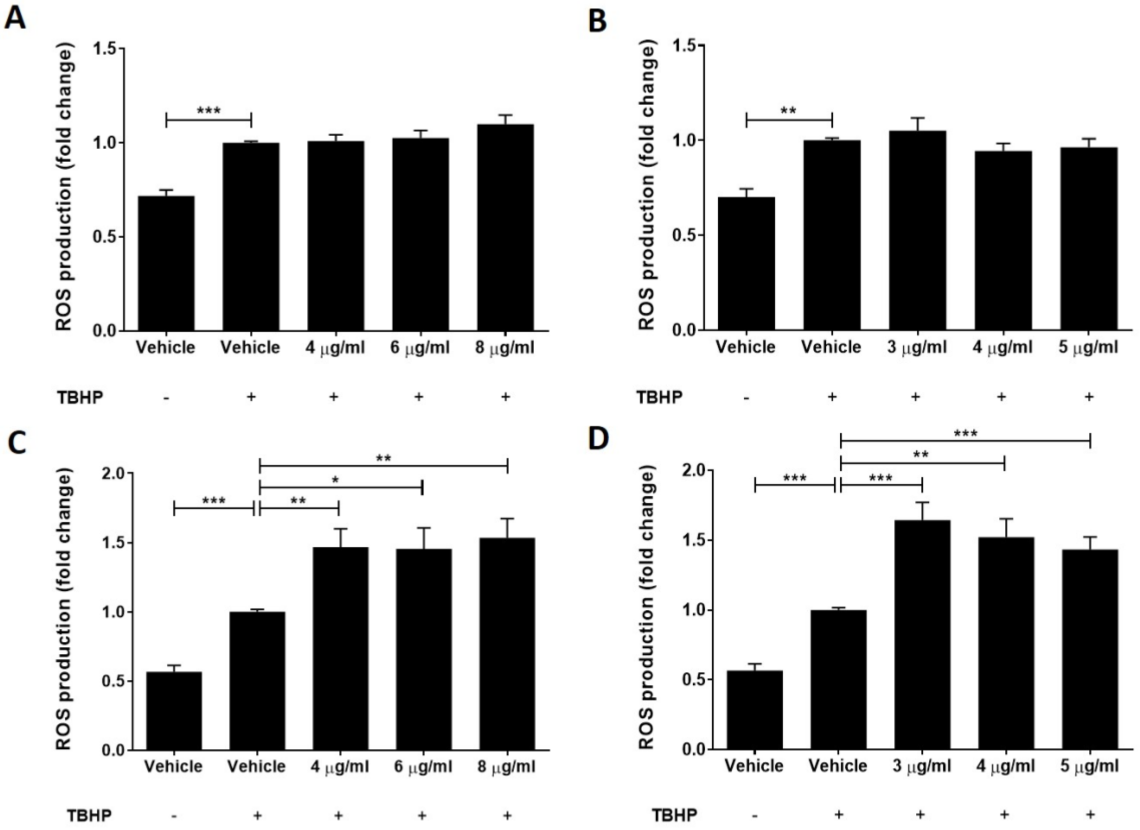

Supplementary Figure 6. Probiotic CM increases TBHP-induced ROS production in human macrophages. TBHP-induced ROS production was determined in THP-1 monocytes **(A-B)** or THP-1 macrophages **(C-D)** following treatment for 3 h with the vehicle control or the indicated concentration of CM from Lab4 **(A, C)** or CUL66 **(B, D)** (TBHP was used at 50 μM). The data are mean ± SEM from five independent experiments (the values from vehicle-treated cells in the presence of TBHP were arbitrarily assigned as 1). Statistical analysis was performed using a one-way ANOVA with Dunnett T3 post-hoc test (* p <0.05, ** p <0.01 and *** p <0.001).
